# Supplementary figures and images for: The JAK-STAT pathway promotes persistent viral infection by activating apoptosis in insect vectors
Source: PLoS Pathog. 2023 Mar 16;19(3):e1011266. doi: 10.1371/journal.ppat.1011266 (PMC10069781; doi:10.1371/journal.ppat.1011266)

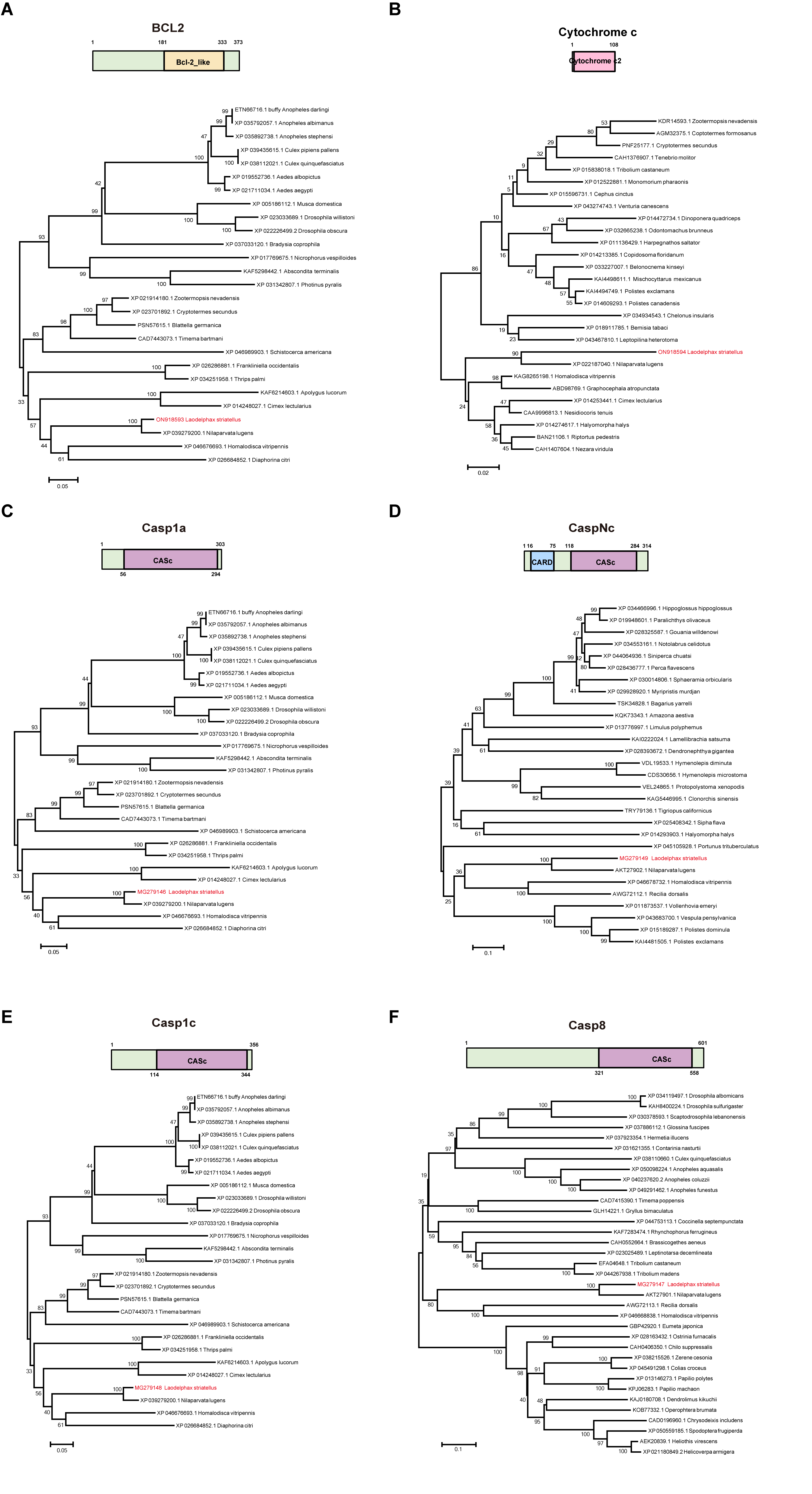

Supplement: S1 Fig — Phylogenetic tree analysis with the maximum likelihood method was based on amino acid sequences of L. striatellus and other insects. (TIF) [file ppat.1011266.s001.tif]

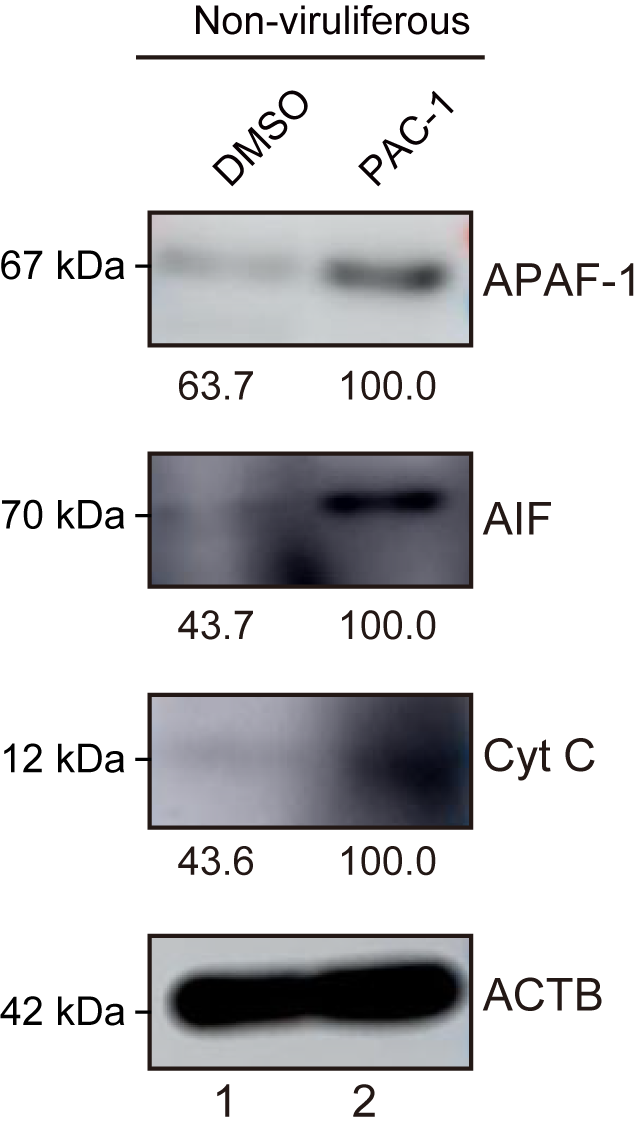

Supplement: S2 Fig — ACTB acted as a protein loading control. (TIF) [file ppat.1011266.s002.tif]

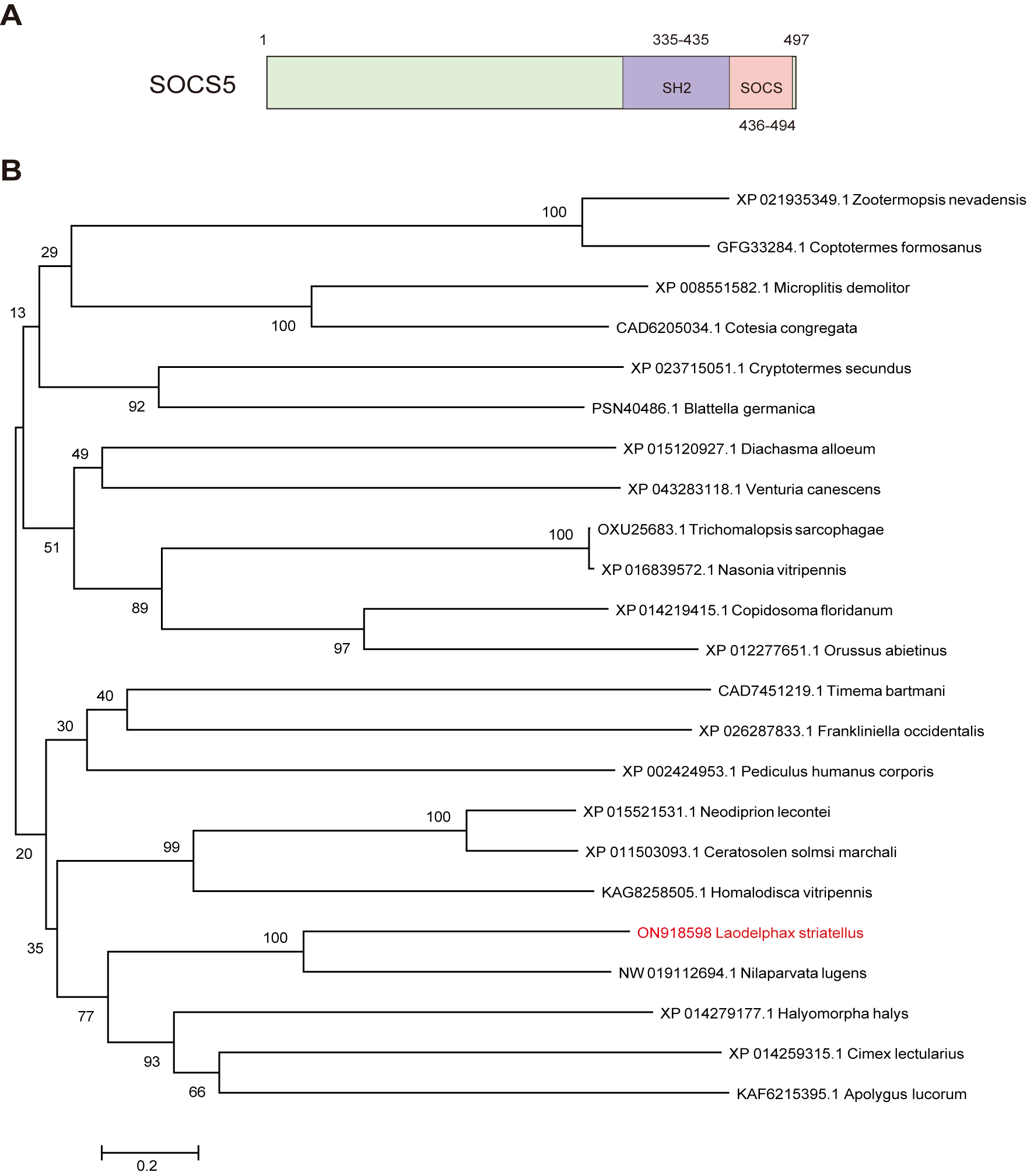

Supplement: S3 Fig — (A) Schematic diagrams showing SOCS5. (B) Phylogenetic tree analysis with the maximum likelihood method was based on amino acid sequences of SBPH and other insects. (TIF) [file ppat.1011266.s003.tif]

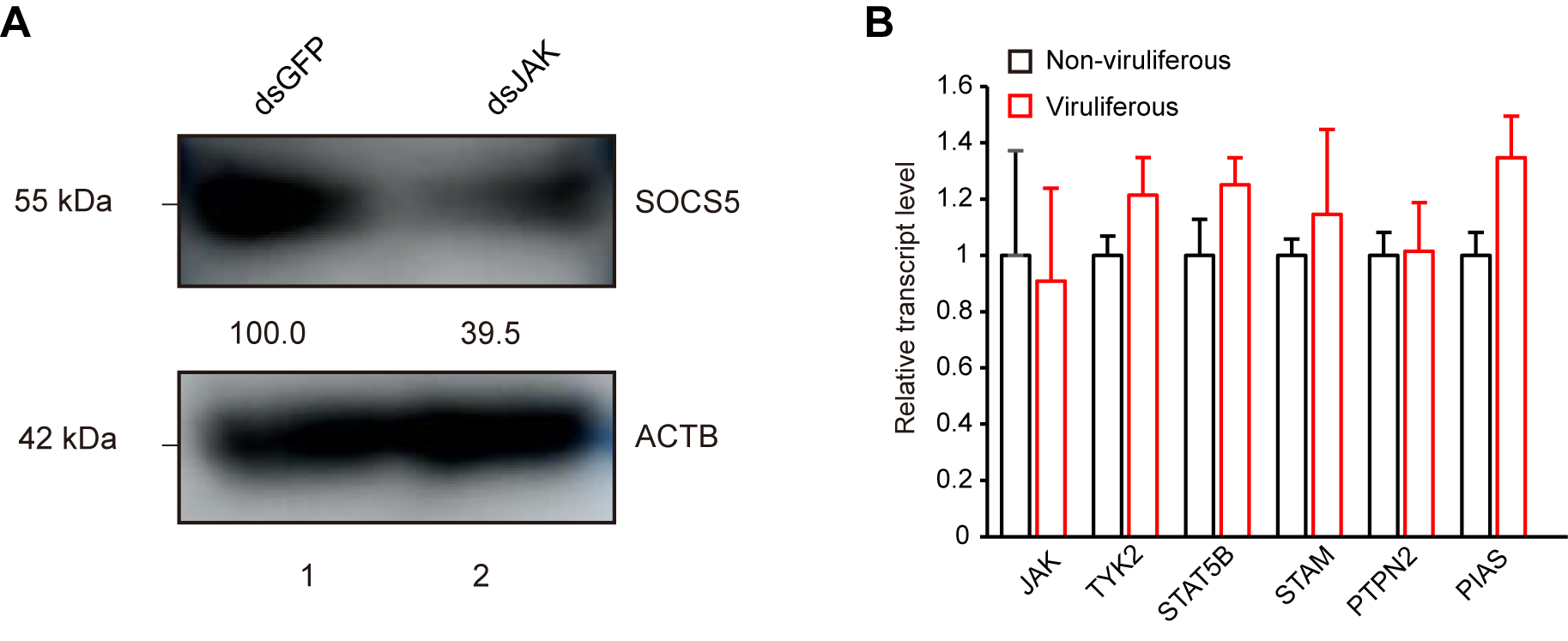

Supplement: S4 Fig — (A) Western blotting analysis of SOCS5 in the dsJAK- or dsGFP-treated nonviruliferous SBPHs. ACTB was used as a protein loading control. (B) Relative transcript expression of JAK-STAT pathway genes in nonviruliferous and viruliferous SBPH as detected by qRT-PCR. Six genes (JAK, TYK2, STAT5B, STAM, PTPN2 and PIAS) from JAK-STAT pathway were detected. The experiment was replicated three times. (TIF) [file ppat.1011266.s004.tif]

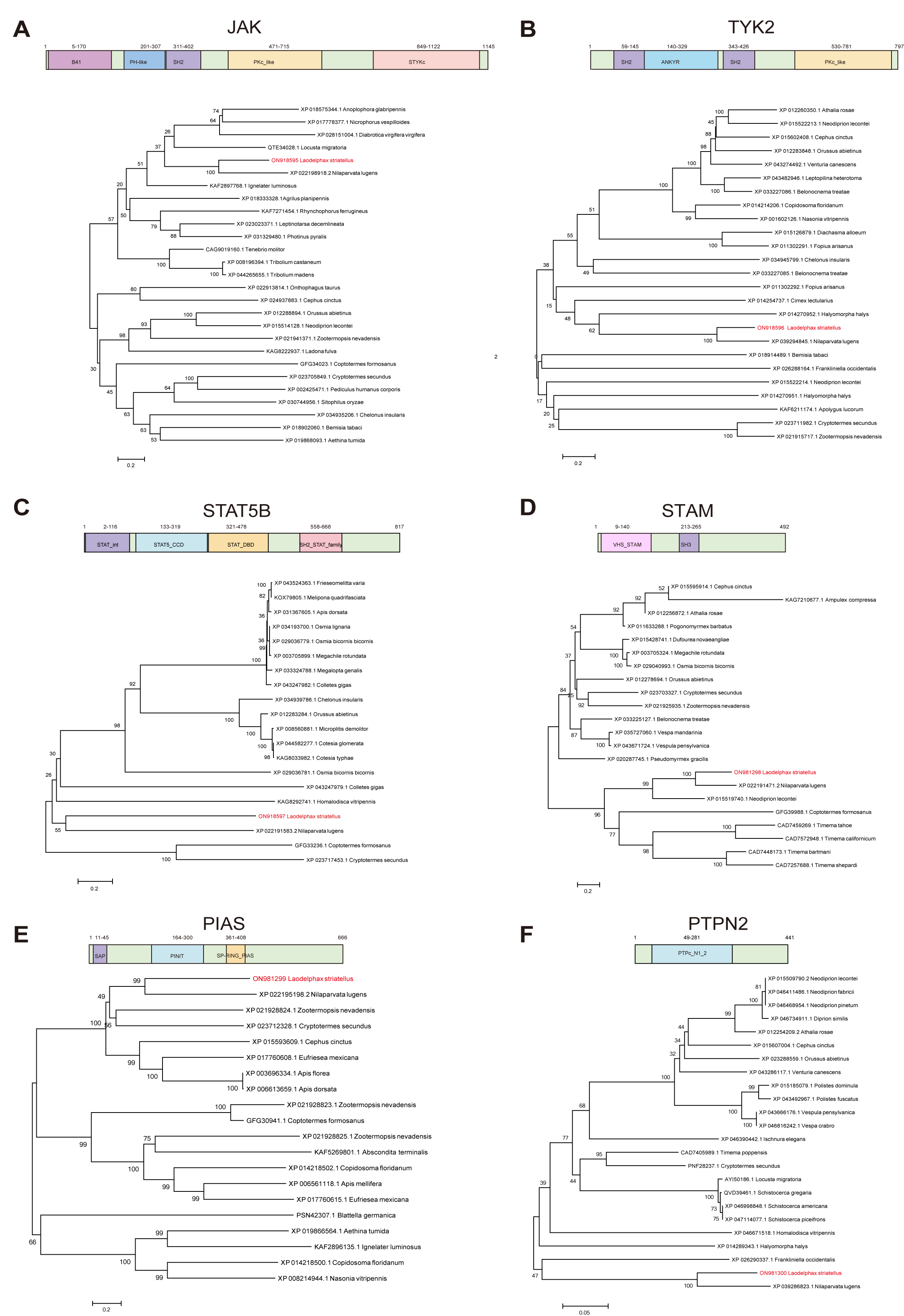

Supplement: S5 Fig — Phylogenetic tree analysis with the maximum likelihood method was based on amino acid sequences of L. striatellus and other insects. (TIF) [file ppat.1011266.s005.tif]

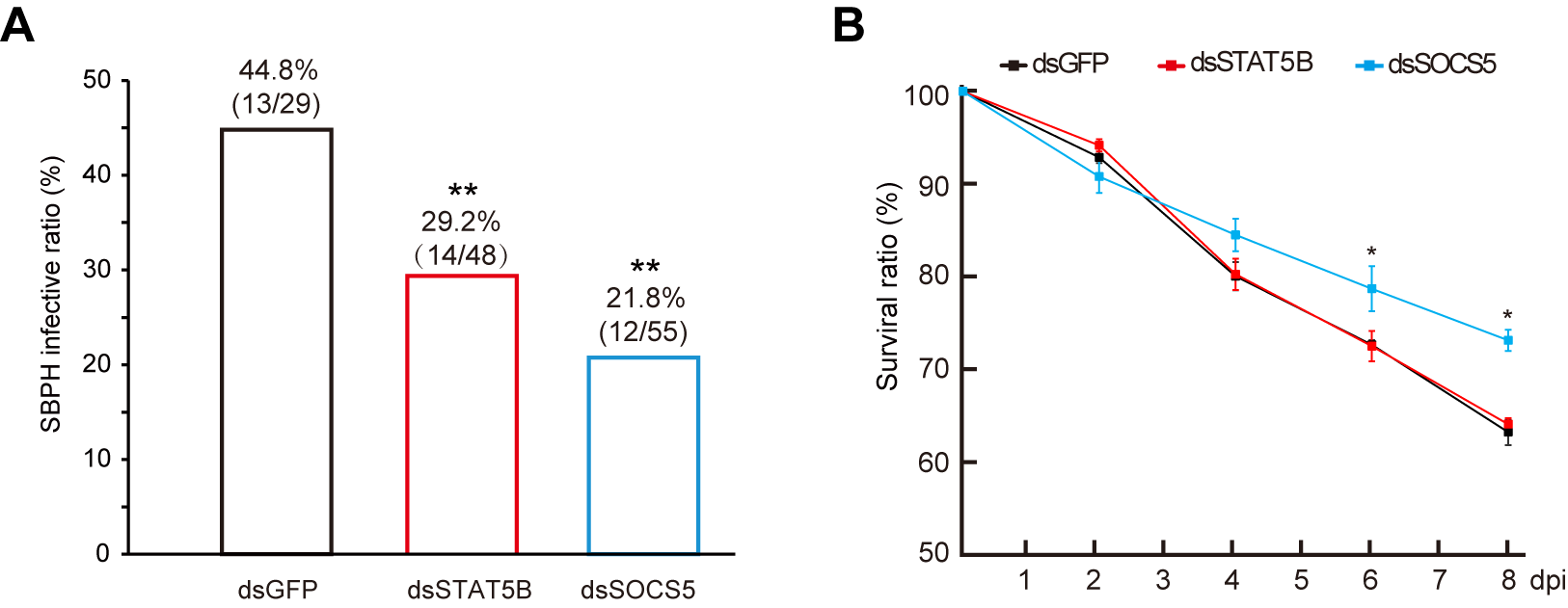

Supplement: S6 Fig — (A) Differences in SBPH infective ratios were compared at 8 days after feeding on RSV-infected rice plants. **, p < 0.01 by the student t-test. (B) Survival of dsGFP-, dsSTAT5B- or dsSOCS5-treated SBPH after the injection of RSV crude extracts. Each point represents the mean value of three biological replicates. *P<0.05 by the student t-test. (TIF) [file ppat.1011266.s006.tif]
