# Supplementary material for: The JAK-STAT pathway promotes persistent viral infection by activating apoptosis in insect vectors
Source: PLoS Pathog. 2023 Mar 16;19(3):e1011266. doi: 10.1371/journal.ppat.1011266 (PMC10069781; doi:10.1371/journal.ppat.1011266)
Supplement: S1 Table — (DOCX) [file ppat.1011266.s007.docx]

**S1 Table. Primers used in this study.**

| **Primer name** | | **Sequence (5' to 3')** | | | | | | |  |  |  |  |  |
| --- | --- | --- | --- | --- | --- | --- | --- | --- | --- | --- | --- | --- | --- |
| RNAi-CaspNC-F | | 5'-GATCACTAATACGACTCACTATAGGGAGAAGAAAAGCGGATCTGGCGAA-3' | | | | | | |  |  |  |  |  |
| RNAi-CaspNC-R | | 5'-GATCACTAATACGACTCACTATAGGGAGAGCCAAACTAGATGCGTTGCC-3' | | | | | | |  |  |  |  |  |
| RNAi-Casp1C-F | | 5'-TAATACGACTCACTATAGGGAGAATGGTCTCGGCAATGGACTG-3' | | | | | | |  |  |  |  |  |
| RNAi-Casp1C-R | | 5'-TAATACGACTCACTATAGGGAGAAGTTGATCTGCGGCACTTGT-3' | | | | | | |  |  |  |  |  |
| RNAi-Casp1-F | | 5'-GATCACTAATACGACTCACTATAGGGAGATCGGAGTATGACTGCCTGGT-3' | | | | | | |  |  |  |  |  |
| RNAi-Casp1-R | | 5'-GATCACTAATACGACTCACTATAGGGAGATAGACCGCGACACGTTGATT-3' | | | | | | |  |  |  |  |  |
| RNAi-Casp8-F | | 5'-TAATACGACTCACTATAGGGAGAAGTGGATCGCCATGGAACTG-3' | | | | | | |  |  |  |  |  |
| RNAi-Casp8-R | | 5'-TAATACGACTCACTATAGGGAGACATGGTAGGCAGTCCGTGTT-3' | | | | | | |  |  |  |  |  |
| RNAi-SOCS5-F | | 5'-GATCACTAATACGACTCACTATAGGGAGACCTGACCCTGTTGTTCCATT-3' | | | | | | |  |  |  |  |  |
| RNAi-SOCS5-R | | 5'-GATCACTAATACGACTCACTATAGGGAGAGAGTTGACTGACCCCATCGT-3' | | | | | | |  |  |  |  |  |
| RNAi-GFP-F | | | | | | 5'-TAATACGACTCACYAYAGGGAGTGGAGAGGGTGAAGG-3' | | | | | | |  |
| RNAi-GFP-R | | | | | | 5'-TAATACGACTCACYAYAGGGAGGGCAGATTGTGTGGAC-3' | | | | | | |  |
| AD-SOCS5-F | | | | | | | 5'-GACGTACCAGATTACGCTCATATGATGGGCCAGAGATTAAGTGATC-3' | | | | | | |
| AD-SOCS5-R | | | | | | | 5'-GCAGCTCGAGCTCGATGGATCCTTACTGCTCGTTGTCAAACC -3' | | | | | | |
| BK-SOCS5-F | | 5'-ATCTCAGAGGAGGACCTGCATATGATGGGCCAGAGATTAAGTGATC-3' | | | | | | |  |  |  |  |  |
| BK-SOCS5-R | | 5'-GCCGCTGCAGGTCGACGGATCCTTACTGCTCGTTGTCAAACC-3' | | | | | | |  |  |  |  |  |
| AD-SOCS5-N-R | | 5'-GCAGCTCGAGCTCGATGGATCCTTAAGGATTGAATTTTGACAAG-3' | | | | | | |  |  |  |  |  |
| AD-SOCS5-C-F | | 5'-GACGTACCAGATTACGCTCATATGATGGAAGACTATCCAATAGAGGA-3' | | | | | | |  |  |  |  |  |
| AD-SOCS5-SH2-F | | 5'-GACGTACCAGATTACGCTCATATGGTGCCTGATCTGCTGCAAAT-3' | | | | | | |  |  |  |  |  |
| AD-SOCS5-SH2-R | | 5'-GCAGCTCGAGCTCGATGGATCCTTAAGGCTCAAAAAACATACAGC-3' | | | | | | |  |  |  |  |  |
| AD-SOCS5-SOCS-F | | 5'-GACGTACCAGATTACGCTCATATGATGCTGACTATCCCGCTG-3' | | | | | | |  |  |  |  |  |
| Pet28a-SOCS5-F | | 5'-CTTTAAGAAGGAGATATACCATGGGCCAGAGATTAAGTGATC-3' | | | | | | |  |  |  |  |  |
| Pet28a-SOCS5-R | | 5'-GTGGTGGTGGTGGTGCTCGAGCTGCTCGTTGTCAAACC-3' | | | | | | |  |  |  |  |  |
| Pet28a-SOCS5-C-F | | 5'-CTTTAAGAAGGAGATATACCATGGAAGACTATCCAATAGAGGA-3' | | | | | | |  |  |  |  |  |
| Pet28a-SOCS5-N-R | | 5'-GTGGTGGTGGTGGTGCTCGAGAGGATTGAATTTTGACAAG-3' | | | | | | |  |  |  |  |  |
| Pet28a-SOCS5-SH2-F | | 5'-CTTTAAGAAGGAGATATACCATGGTGCCTGATCTGCTGCAAAT-3' | | | | | | |  |  |  |  |  |
| Pet28a-SOCS5-SH2-R | | 5'-GTGGTGGTGGTGGTGCTCGAGAGGCTCAAAAAACATACAGC-3' | | | | | | |  |  |  |  |  |
| Pet28a-SOCS5-SOCS-F | | 5'-CTTTAAGAAGGAGATATACCATGCTGACTATCCCGCTG-3' | | | | | | |  |  |  |  |  |
| MBP-BCL2-F | | 5'-CTGTATTTTCAGGGCCATATGATGTCGGATTCAAAAGCG-3' | | | | | | |  |  |  |  |  |
| MBP-BCL2-R | | 5'-ACGGAGCTCGAATTCGGATCCTTAAAGCGGCGCAAACTTTC-3' | | | | | | |  |  |  |  |  |
| RNAi-STAT5B-F | | 5'-TAATACGACTCACTATAGGGAGAACCGCCTCAAGTGATGAAGA-3' | | | | | | |  |  |  |  |  |
| RNAi-STAT5B-R | | 5'-TAATACGACTCACTATAGGGAGAGATCGGGCACAAAGAACGGC-3' | | | | | | |  |  |  |  |  |
| Pet28a-STAT-F | | 5'-CTTTAAGAAGGAGATATACCATGAGTTTGTGGGCGAAAAC-3' | | | | | | |  |  |  |  |  |
| Pet28a-STAT-R | | 5'-GTGGTGGTGGTGGTGCTCGAGGTTAAGTTGGTTGTTGTTG-3' | | | | | | |  |  |  |  |  |
| SBS1-F | | 5'-CGTGCCACTCTATCGTGGTT-3' | | | | | | |  |  |  |  |  |
| SBS1-R | | 5'-TGACCCTACAAACTGGCAACA-3' | | | | | | |  |  |  |  |  |
| SBS2-F | | 5'-TGACCAGGAACAATGCCCTA-3' | | | | | | |  |  |  |  |  |
| SBS2-R | | 5'-GCCAGAGCTAAGTCCCGAAC-3' | | | | | | |  |  |  |  |  |
| SBS3-F | | 5'-CACAGAAAATTCAACGTCATCTTCA-3' | | | | | | |  |  |  |  |  |
| SBS3-R | | 5'-TGACTTCAAGACCTGCCACA-3' | | | | | | |  |  |  |  |  |
| pABAi-SBS1-F | | 5'-AAGCTTGAATTCGAGCTCCGTGCCACTCTATCGTGGTT-3' | | | | | | |  |  |  |  |  |
| pABAi-SBS1-R | | 5'-GCACATGCCTCGAGGTCGACTGACCCTACAAACTGGCAACA-3' | | | | | | |  |  |  |  |  |
| pABAi-SBS2-F | | 5'-AAGCTTGAATTCGAGCTCTGACCAGGAACAATGCCCTA-3' | | | | | | |  |  |  |  |  |
| pABAi-SBS2-R | | 5'-GCACATGCCTCGAGGTCGACGCCAGAGCTAAGTCCCGAAC-3' | | | | | | |  |  |  |  |  |
| pABAi-SBS3-F | | 5'-AAGCTTGAATTCGAGCTCCACAGAAAATTCAACGTCATCTTCA-3' | | | | | | |  |  |  |  |  |
| pABAi-SBS3-R | | 5'-GCACATGCCTCGAGGTCGACTGACTTCAAGACCTGCCACA-3' | | | | | | |  |  |  |  |  |
| AD-STAT-F | | 5'-TACCAGATTACGCTCATatgATGAGTTTGTGGGCGAAAAC-3' | | | | | | |  |  |  |  |  |
| AD-STAT-R | | 5'-GCAGCTCGAGCTCGATGGATCCTTAGTTAAGTTGGTTGTT-3' | | | | | | |  |  |  |  |  |
| q-CaspNc-F | | 5'-AGCGCTTTAGCCTTCACAGA -3' | | | | | | |  |  |  |  |  |
| q-CaspNc-R | | 5'-GTAGCCCTTCGGTTTCGAGT -3' | | | | | | |  |  |  |  |  |
| q-Casp1C-F | | 5'-GCGTTCAGATTCAGTGCGTC-3' | | | | | | |  |  |  |  |  |
| q-Casp1C-R | | 5'-TGTACACTCGGGCACACTTC-3' | | | | | | |  |  |  |  |  |
| q-Casp1-F | | | 5'-AAAACCAAAAGCGAACGGGG -3' | | | | | | |  |  |  |  |
| q-Casp1-R | | | 5'-GAAAATTATCGCCAGGCCGC -3' | | | | | | |  |  |  |  |
| q-Casp8-F | | | | 5'-AGTGGATCGCCATGGAACTG-3' | | | | | | |  |  |  |
| q-Casp8-R | | | | 5'-GTGTTGCTTGAATTCGCGGT-3' | | | | | | |  |  |  |
| q-SOCS5-F | | | | | 5'-CCGGTAAGTCGTGTCCGAAA -3' | | | | | | |  |  |
| q-SOCS5-R | | | | | 5'-GCAAACACACGAGTCACACC -3' | | | | | | |  |  |
| q-STAT5B-F | | 5'-CGAGCGACTGAAGGACACAA-3' | | | | | | |  |  |  |  |  |
| q-STAT5B-R | | 5'-CTGCCGGTTCAGCCAGATTA-3' | | | | | | |  |  |  |  |  |
| q-actin-F | | 5'-AGTGCCCATCTACGAAGGTTACG-3' | | | | | | |  |  |  |  |  |
| q-actin-R | | 5'-CGGCGGTGGTGGTGAAGC-3' | | | | | | |  |  |  |  |  |
| q-RSV-CP-F | | 5'-TGCAGAAGGCAATCAATGACAT-3' | | | | | | |  |  |  |  |  |
| q-RSV-CP-R | | 5'-TGTCACCACCTTTGTCCTTCAA-3' | | | | | | |  |  |  |  |  |
| q-RB-P10-F | | 5'-AACAACCGACCAACAATCAC-3' | | | | | | |  |  |  |  |  |
| q-RB-P10-R | | 5'-GAGCAGGAACTTCACGACAG-3' | | | | | | |  |  |  |  |  |
