# Supplementary material for: The JAK-STAT pathway promotes persistent viral infection by activating apoptosis in insect vectors
Source: PLoS Pathog. 2023 Mar 16;19(3):e1011266. doi: 10.1371/journal.ppat.1011266 (PMC10069781; doi:10.1371/journal.ppat.1011266)
Supplement: S2 Table — (DOCX) [file ppat.1011266.s008.docx]

**S2 Table. The detailed information of commercial antibodies used in this study**

| **Proteins** | **Predicted band size (kDa)** | **Company** | **Item No.** | **UniProt No.** | **Amino acid identities** |
| --- | --- | --- | --- | --- | --- |
| BCL2 | 26 | Huabio | Rabbit, ET1702-53 | Human, P10415 | 26% |
| Cyt C | 12 | Huabio | Rabbit, R1510-41 | Human, P99999 | 73% |
| AIF | 70 | Huabio | Rabbit, ET1603-4 | Human, O95831 | 56% |
| Apaf-1 | 67 | Huabio | Rabbit, ET1607-12 | Human, O14727 | 24% |
| P-STAT5B | 100 | Cell Signaling Technology | Rabbit, 9351 | Human, P51692 | 44% |
| SOCS5 | 55 | Huabio | Rabbit, HA500307 | Human, Q8WXH5 | 70% |
