# Supplementary material for: The JAK-STAT pathway promotes persistent viral infection by activating apoptosis in insect vectors
Source: PLoS Pathog. 2023 Mar 16;19(3):e1011266. doi: 10.1371/journal.ppat.1011266 (PMC10069781; doi:10.1371/journal.ppat.1011266)
Supplement: Attachment — Submitted filename: Response to reviewers.docx [file ppat.1011266.s009.docx]

**Response to Reviewers:**

*Reviewer #1: In this study Zhang et al. report that activation of JAK/STAT pathway by RSV or RBSDV infection promotes virus accumulation in their insect vectors through positively regulation of the apoptosis pathway. More specifically, the authors show that SOCS5, a negative regulator of JAK/STAT pathway, directly interacts with BCL2 and accelerates its degradation. Decreased inhibition by BCL2 induces apoptosis, which enhance virus replication in SBPH. This study is innovative, but some data is not enough to support the author's conclusions. The method used to compare the amount of virions in salivary glands of planthoppers is inappropriate. Besides, there are many grammatical mistakes in this manuscript. The authors need resolve these questions before publication.*

**Response: We thank the reviewer #1 for his/her great comments and suggestions.** **We have taken time to revise our manuscript to make the conclusion clearer. We have replaced with relative intensity ratio (RSV-NP/DAPI) to compare the amount of virions in planthoppers (See new Fig 2L and 2M, Fig 5G and 5H). We also have modified the language of the manuscript to make it suitable for publication.**

*Reviewer #2: In this manuscript authors described the role of JAK-STAT pathway in promoting persistent viral infection by activating apoptosis in insect vectors small brown planthopper. They showed that both RSV and RBSDV, two important rice viruses transmitted by small brown planthopper, activated the JAK-STAT pathway and promoted the accumulation of Suppressor of cytokine signaling 5 (SOCS5), an E3 ubiquitin ligase regulated by the JAK-STAT transcription factor STAT5B. They showed strong evidence that SOCS5 could directly interact with the anti-apoptotic B-cell lymphoma-2 (BCL2) to accelerate the BCL2 degradation through the 26S proteasome pathway, thus activating apoptosis and facilitating persistent viral infection in their vector by an unknown mechanism. The manuscript includes a large amount of data supporting the conclusions drawn and the findings are very interesting. Overall, the authors present plenty of interesting observations that directly validate that they do by combining molecular and cell biology, biochemistry approaches. However, the shortcoming of this manuscript is the lack of replicates in many experiments. I would recommend acceptance after responding to these concerns. I have outlined my specific concerns and suggestions below.*

**Response: We also thank reviewer#2 for his/her great comments and encouragement.**

*Reviewer #3: Zhang et al provided a story about virus-induced JAK-STAT pathway of insect vector regulates apoptosis to promote viral infection. They found that both RSV and RBSDV infection activated the JAK-STAT pathway and promoted the accumulation of SOCS5, which was regulated by the STAT5B. Virus-induced SOCS5 interacted with BCL2 to accelerate the BCL2 degradation through the 26S proteasome pathway. As a result, the activation of apoptosis facilitated persistent viral infection in their vector.*

*This paper presents an interesting story about virus-vector interaction. However, there are many overstatements in interpretation/conclusions of main finding regarding the occurrence of apoptosis, and the association between virus infection and apoptosis. It is essential that the authors clearly explain the apoptotic phenotypes, enzymology and pathway, the way to viral acquisition of insect, as well as the biological role of the main proteins of SBPH. In the absence of such clarifications, it is overreaching to conclude that JAK-STAT pathway promotes viral infection by activating apoptosis in insect vectors.*

**Response: We thank reviewer#3 for his/her comments and criticism. In the past few months we have tried our best to add more new data to confirm our conclusions. We hope that this new manuscript meets the requirements of PLOS Pathogens.**

*Reviewer #1: 1. It is believed that SOCS family are the negative-feedback regulators of JAK/STAT pathway. However, the protein levels of phosphorylated STAT5B did not have an obviously increase after dsSOCS5 treatment in Fig. 5K. In many cases, the transcriptional activation of SOCS genes is mediated by the STAT proteins. The authors did not provide the experimental evidence to further demonstrate the relationship between small brown planthopper JAK/STAT pathway and SOCS5. Thus, it is incorrect to conclude that JAK/STAT pathway regulate the apoptosis pathway via SOCS5.*

**Response: Thanks for your good suggestions. Indeed, SOCS5 acts as a negative regulator to affect the phosphorylation level of STAT5B. However, other negative regulators (e.g. PIAS, TC-PTP, SHP1) of JAK/STAT pathway may affect the phosphorylation level of STAT5B after dsSOCS5 treatment. In addition, it is not ruled out the possibility that viral proteins may also be involved in regulating the phosphorylation level of STAT5B. Thus, the protein levels of phosphorylated STAT5B showed a slight increase after dsSOCS5 treatment (new Fig 5K).**

**We have predicted three putative STAT5B binding sites (SBS1, SBS2 and SBS3) in the SOCS5 promoter (new Fig 4A). Further, we have also performed yeast one-hybrid assay and electrophoretic mobility shift assay (EMSA) to confirm that the SOCS5 promoter region could be targeted by STAT5B protein (new Fig 4B and 4C). In addition, the protein levels of SOCS5 were significantly reduced after dsSTAT5b treatment (new Fig 5K). In sum, our results showed that the transcriptional activation of *SOCS5* was mediated by the STAT5B protein.**

*2. Fig. 3E: after treated with proteasome inhibitor, the protein levels of BCL2 obviously increased compared with the controls. But the amount of SOCS5 was lower in inhibitor-treated SBPHs than in the controls. It can’t exclude the effect of SOCS5 reduction on inhibition of BCL2 degradation.*

**Response: We agree with this comment and have reconducted the inhibitor treatment experiment. The results showed that the protein levels of SOCS5 did not significantly decrease compared with the controls. We also performed the inhibitor treatment experiment in non-viruliferous planthoppers and showed the similar results (new Fig 3E). Therefore, these results suggested that inhibitor treatment did not affect on the protein levels of SOCS5.**

*3. The description of some results is incomplete. i.e. Fig. 3E. The authors did not mention the effect of inhibitor treatment on the protein levels of SOCS5. The manuscript requires a thorough revision because it contains a high number of inaccuracies and minor problems.*

**Response: We have now modified the text based on this comment (Lines 197-201).** **We also recognized our issues in manuscript writing and have tried our best to modify the text to meet the requirements of PLOS Pathogens.**

Reviewer #2: Specific concerns:

*1. Figure 1F needs to verify this observation repeatedly, and no replicates can be found in the figure. In addition, Figure 1A needs strict statistics, how many/what is the percentage of virus-infected epithelial cells displaying condensed nuclei and marginalized chromatin?*

**Response: We have performed three repetitions (Rep1, Rep2 and Rep3) in the immunoblotting assays (new Fig 1G). We have also added the statistical analysis for apoptotic events in non-viruliferous and viruliferous planthoppers (new Fig 1B).**

*2. Figure 2 the authors should confirm that after using RNA-mediated interference (RNAi) technology to reduce the expression of four apoptosis factors, cell apoptosis is indeed disturbed. This is the important control.*

**Response: We appreciate the helpful comments and the protein levels of cytochrome *c* (Cyt C) were taken as an indicator of apoptotic event. The results showed that the protein levels of Cyt C obviously decreased after the four apoptosis factors were reduced (new Fig 2I).**

*3. In Figure 4, in the viruliferous SBPH, phospho-STAT5 has two bands whereas in non-viruliferous control, not a band could be found. Please explain this phenomenon.*

**Response: We have re-performed three repetitions to detect the protein levels of phosphorylated STAT5B (P-STAT5B) in non-viruliferous and viruliferous planthoppers (new Fig 4D). Similar, the protein levels of P-STAT5B were still difficult to detect in non-viruliferous control. Meanwhile, The predicted band of P-STAT5B (100 kDa) could be observed in viruliferous samples and another bands were considered as a nonspecific band.**

*Reviewer #3: 1. The most important problem is demonstration of apoptotic events. In general, the measurements of apoptotic events contain cell morphology like abnormal nuclei and apoptotic bodies, enzymology like cleavage of caspase, and activation of pathways like mitochondrial pathway. This study appears insufficient of evidences for apoptotic events, due to the unclear TEM images, absence of caspase cleavage data, and the limited investigation on mitochondrial pathway. Just providing TUNEL data is unsolid to verify the occurrence of apoptosis.*

**Response: We carefully considered this reviewer’s suggestions and comments. We have performed several experiments to support the apoptotic events in viruliferous planthoppers: 1) We have added the statistical analysis for apoptotic events based on the observation of TEM images and have found that the apoptotic cells were significantly highly in viruliferous than these in non-viruliferous samples (new Fig 1B); 2) We have immunolabeled RSV virion in the TUNEL assays to support that the apoptotic cells was induced by RSV infection (new Fig 1C-1F); 3) We have performed immunoblotting assays using two key factors of mitochondrial-dependent apoptosis (APAF-1 and AIF) to detect the apoptotic events during viral infection (new Fig 1G and 1H).**

*1) In TEM (Fig. 1A), the cellular profiles were incomplete, which made it difficult to distinguish the occurrence of nuclei condense and chromatin marginalization. The mitochondrial cristae in cells of nonviruliferous SBPHs were unclear. While the mitochondrial cristae in cells of viruliferous SBPHs were clear, and mitochondria did not show vacuolation. The characterization on lines 108-112 are different from what Fig.1A showed. More important, how did authors confirm that those cells were RSV-infected, because there was no virus or virus-induced inclusion body in the cells of viruliferous SBPHs.*

**Response: We have replaced these TEM images (new Fig 1A). For confirm that apoptotic cells were RSV-infected, we have immunolabeled RSV virion in the TUNEL assays and have performed immunoblotting with antibody against RSV NP. These viruliferous samples were all from the same population.**

*2) In the TUNEL assays of midgut and salivary glands, immunolabel of virus was required to prove that apoptosis was induced by virus infection. In addition, the DAPI label showed the absence of crescent-shaped nuclei, which was the typical morphology of apoptosis, in the midgut and salivary glands of viruliferous SBPHs.*

**Response: Thank you for this suggestion. We have added the immunolabel of virus in the TUNEL assays and the results showed that the majority of apoptotic cells contained RSV virions (new Fig 1C and 1E). We have also included this description in the text (Lines 122-123).**

*3) The basic enzymology of apoptotic events was cleavage of caspase. In results section, authors did not show the caspase expression and cleavage. They just and sometimes showed the expression of CytC or BCL2, which is totally overstated for the occurrence of apoptosis. Furthermore, when mitochondrial-dependent apoptosis takes place, CytC releases from mitochondrial interior to cytoplasm, causing the increased level of CytC in cytoplasm. Authors are suggested compare the CytC accumulation in purified mitochondria and cytoplasm of viruliferous and nonviruliferous SBPHs.*

*In addition, authors are suggested analyze key factors of mitochondrial-dependent apoptosis during viral infection, such as AIF, Smac/DIABLO, APAF-1, caspase9, and so on.*

**Response: We agree these comments and suggestions. However, we were unable to find a suitable antibody to detect cleavage of caspase 3. Instead, we have detected protein levels of several key factors (i.e. CytC, BCL2, APAF-1 and AIF) during viral infection (new Fig 1G and 1H). Our results showed that apoptotic events were indeed occurred during RSV infection.**

*4) A positive control of apoptosis event was required to show the mitochondrial cristae, nucleic, and the accumulation of CytC and BCL2.*

**Response: We have used treatment of apoptotic activator (PAC-1) to non-viruliferous planthoppers as a positive control (new S2 Fig).**

*5) Lines 135-140, if the accumulation of CytC and BCL2 and TUNEL results were taken as the apoptotic indicators, they should also be shown here.*

**Response: CytC was taken as the apoptotic indicator and we have added these contents (Line 155).**

*6) When the apoptotic inhibitor or activator was used to test the effect of apoptosis on viral infection, the effect of inhibitor or activator on apoptosis was also required to show.*

**Response: We have done so (new Fig 2J)**

*7) Fig.2K and Fig. 5F, why were salivary glands preferred to be evidences for apoptotic organs, but not midgut or ovary? Did salivary glands represent intact insects?*

**Response: Thanks for your good suggestions. RSV not only spreads to the salivary glands but also spreads to the reproductive system. We have added ovary organs to observation of the effects of apoptosis on viral infection (new Fig 2K and Fig 5F).**

*2. Another main problem is the way to viral acquisition of insect. Most ways are hemolymph injection with virus crude extracts from viruliferous SBPHs in this study.*

*1) Why was this way applied, for example line 122? I think hemolymph injection is also a way to transfer the immune signal of insect by human, or secondary activation of the immune signal. Did apoptosis occur in virulifeorus SBPHs via oral acquisition of virus?*

**Response: Hemolymph injection was one way of viral acquisition and this way had an obviously increase of viral acquisition rate compared to oral acquisition. To avoid activating immune signals, mock injection (hemolymph injection with virus-free crude extracts from non-viruliferous SBPHs) was included as a control in each experiment and our results showed that apoptotic events were not induced by mock injection (new Fig 1H). In addition, apoptosis was also occurred in virulifeorus SBPHs via oral acquisition of virus.**

*2) Lines 135-136, Were insects injected with dsRNAs and virus crude extracts injected together? Or injection with dsRNAs advanced for virus crude extracts?*

**Response: We are sorry for this confusion. We injected with dsRNAs advanced for virus crude extracts.**

*3) Lines 214-215, how did SBPHs acquire virus? Oral acquisition of virus or hemolymph injection with dsRNAs? I do not understand the biological significance of testing the capability of RSV acquisition.*

**Response: SBPH acquired virus using hemolymph injection after 2 days post infection with dsRNAs (Lines 265-267). Hemolymph injection could mimic oral viral acquisition and could be used to investigate the effect of JAK-STAT pathway on viral infection. We also conducted oral acquisition assay that SBPH were fed to RSV-infected rice samples for 48h after 2 days post infection with dsRNAs (new S6A Fig). Similar to the results of hemolymph injection of RSV crude extracts, inhibition the expression of STAT5B or SOCS5 also significantly reduced RSV acquisition by SBPHs.**

*3. The biological role of the key proteins of SBPHs were not verified.*

*1) The biological function of SOCS5 and STAT5B in SBPHs were unclear. Authors should prove the association of SOCS5 with apoptosis, including the effect of RNAi targeting SOCS5 on BCL2 accumulation and apoptotic level. Authors should also prove the role of STAT5B in JAK-STAT pathway, including the effect of RNAi targeting STAT5B on apoptotic level and accumulation of other factors of JAK-STAT pathway.*

**Response: The protein levels of BCL2, CytC, APAF-1 and AIF were detected after injection of dsRNA-*SOCS5* and dsRNA-*STAT5B* (new Fig 5K)**

*2) Line 168-171, these results only proved SOCS5 negatively regulated BCL2, but not SOCS5 degraded BCL2.*

**Response: Thanks for point it out. We have rephrased the text (Lines 194-195)**

*3) Lines 205-217, these results only proved the association of STAT5B or SOCS5 with RSV infection and genomic replication, but did not show the relationship between STAT5B and SOCS5.*

**Response: We have predicted three putative STAT5B binding sites (SBS1, SBS2 and SBS3) in the SOCS5 promoter (new Fig 4A). Further, we have also performed yeast one-hybrid assay and electrophoretic mobility shift assay (EMSA) to confirm that the SOCS5 promoter region could be targeted by STAT5B protein (new Fig 4B and 4C). In addition, the protein levels of SOCS5 were significantly reduced after dsSTAT5b treatment (new Fig 5K). In sum, our results showed that the transcriptional activation of *SOCS5* was mediated by the STAT5B protein.**

*Other comments:*

*1. In the 26S proteasome assay, the treatment of MG132 only showed that absence of 26S proteasome system affected RSV infection, accumulation of BCL2 and SOCS5, but did not show the relationship among BCL2, SOCS5 and RSV infection. Authors should provide data that response of 26S proteasome system to RSV infection, and to RNAi targeting SOCS5.*

**Response: We have added the experiment that MG132 treatment in nonviruliferous SBPHs had no effect on the accumulation of BCL2 and SOCS5 (new Fig 3E). We have also performed mitochondria isolation assay and the results showed that both of the two proteins (BCL2 and SOCS5) were mainly found in the mitochondria, but not in cytosol. During RSV infection, the accumulation of BCL2 significantly decreased and an obvious accumulation of SOCS5 was detected in mitochondria (new Fig 3F).**

*Did MG132 treatment in nonviruliferous SBPHs affect accumulation of BCL2 and SOCS5?*

**Response: Compared with the control, there was no effect on the accumulation of BCL2 and SOCS5 after MG132 treatment in nonviruliferous SBPHs (new Fig 3E).**

*Why did authors think that SOCS5-induced reduction of BCL2 protein was 26S proteasome system dependent?*

**Response: 26S proteasome system was involved, but not dependent in the reduction of BCL2 protein.**

*2. Authors found that virus infection only affected the accumulation of P-STATB, but had no effect on other key genes of JAK-STAT pathway. It was unreasonable to conclude that virus infection activated the JAK-STAT pathway. I think virus infection just activated P-STATB.*

**Response: Virus infection promoted the accumulation of SOCS5, which was mediated by the STAT5B protein (new Fig 4). When we injected dsRNAs to reduce the expression of JAK in viruliferous SBPHs, the protein levels of SOCS5 were obviously decreased (new S4A Fig). Our results showed that not only STAT5B, but also other genes in the JAK-STAT pathway regulated the expression of SOCS5 protein during virus infection.**

*3. Fig. 4, qPCR assays showed that virus could be detected at 2 and 4 dpi, but the mRNA of SOCS5 and STAT5B did not show notable change. These results indicated that the viral propagation did not activate more SOCS5 and STAT5B expression. Therefore, did most P-STAT5B and SOCS5 detected by western blots derive from hemolymph injection of virus crude extracts?*

**Response: We have addressed this issue: 1) Although no notable change was shown at mRNA levels, viral infection might somehow inhibit the protein degradation of SOCS5 and STAT5B; 2) We collected 30 SBPHs per time point for western blot analysis and the two proteins derived from virus crude extracts were hardly detected by western blots; 3) the protein levels of P-STAT5B and SOCS5 were higher in viruliferous than in non-viruliferous SBPHs (new Fig 4D). Thus, P-STAT5B and SOCS5 detected by western blots did not derive from the crude extracts, but activated by viral infection.**

*Western blots showed that when virus could not be detected, P-STAT5B accumulated at a high level. This result could not prove that virus activated P-STAT5B.*

**Response: Although virus could not be detected by western blots at 2 dpi, qRT-PCR assays showed that a significant increase of viral accumulation was detected at 2 dpi (new Fig 4F). Our results supported that viral infection activated P-STAT5B.**

--------------------

*<b>Part III – Minor Issues: Editorial and Data Presentation Modifications</b></br>Please use this section for editorial suggestions as well as relatively minor modifications of existing data that would enhance clarity.<br/><br/>*

*Reviewer #1: 1. Line 19 Define all abbreviations at first mention in the text.*

*2. Line 26 The letter s does not need to be capitalized.*

*3. Line 85 “Infection” is an uncountable noun when it means the act or process of causing or getting a disease.*

*4. Line 122 RSV crude extracts from viruliferous SBPHs?*

*5. Line 231 The statement “the expression of P-STAT5B and SOCS5 were significantly increased and BCL2 was reduced remarkably” is incorrect. The authors detected the protein levels of P-STAT5B, SOCS5 and BCL2, but not mRNA levels of these genes.*

**Response: We thank the reviewer for these suggestions. We have corrected these problems accordingly.**

*6. Fig. 2L and 5G: how many salivary glands of SBPHs were used to calculate the proportions of RSV-infected cells in salivary glands? Usually, the number of virions differ greatly in different virus-infected cells. Why not analyze the fluorescence density of virus in salivary glands?*

**Response: 30 salivary glands per treatment were used to calculate the proportions of RSV-infected cells and we have used relative fluorescence intensity to calculate the proportions (new Fig 2L and 2M, Fig 5G and 5H).**

*7. Fig 3D: How do the authors explain that SOCS5 promotes the degradation of BCL2 mainly in the presence of virus? Is it possible that viral proteins are involved in the process?*

**Response: During the absence of virus, the expression of SOCS5 was at a low level and had no effect on the BCL2 accumulation. Virus infection activated JAK-STAT pathway and promoted SOCS5 accumulation. The highly expressed SOCS5 interacted with BCL2 and accelerated BCL2 degradation. Also, it cannot be ruled out the possibility that SOCS5 was targeted by viral proteins to promote BCL2 degradation.**

*8. Fig. 4B and 6A: Are viruliferous and nonviruliferous insect samples in the same PDVF membrane? If not, the authors cannot compare the difference between them.*

**Response: These samples are in the same PVDF membrane.**

*9. I think inclusion of an in vivo experiment to validate the interaction between SOCS5 and BCL2 in SBPHs would be better, such as coimmunoprecipitation, immuno-colocalization.*

**Response: We have also performed mitochondria isolation assay and the results showed that both of the two proteins (BCL2 and SOCS5) were mainly found together in the mitochondria, but not in cytosol (new Fig 3).**

*Reviewer #2: Some other suggestions:*

*1. The writing of this manuscript needs improved.*

*2. Page 5, line 88, “JNK” should be described in detail as it appears for the first time in the manuscript. The authors should correct this mistake throughout the manuscript. For example, lines 98, 112…*

*3. The commercial antibodies used in this manuscript, such as anti-Phospho-STAT5, anti-BCL2 and so on are mostly designed for mice and human. Authors should describe the amino acid sequence similarity between mice/human.*

**Response: We have found the mistakes and have corrected. We shall try our best to improve our manuscript writing skill. We have also described the amino acid sequence similarity (new S2 Table).**

*Reviewer #3: 1. Lines 131-133, what are CaspaseNC, Traf6 and dMiro? The function of these proteins playing in apoptosis events should be mentioned in introduction.*

**Response: These proteins have been mentioned in the result part (Lines 148-150).**

*2. “Artificial double parafilm feeding” should appear in lines 140-141 for the first time.*

**Response: We have revised it (Line 160).**

*3. Line 216, what did “nonviruliferous SBPHs during RSV infection” mean?*

**Response: We have revised this sentence (Lines 271-272).**
