# Supplementary material for: The JAK-STAT pathway promotes persistent viral infection by activating apoptosis in insect vectors
Source: PLoS Pathog. 2023 Mar 16;19(3):e1011266. doi: 10.1371/journal.ppat.1011266 (PMC10069781; doi:10.1371/journal.ppat.1011266)
Supplement: Attachment — Submitted filename: Response to reviewers-2-27-2023.docx [file ppat.1011266.s010.docx]

**Response to Reviewers:**

*Reviewer #1: In this study the authors described the role of JAK-STAT pathway in the persistent viral infection in small brown planthopper. They showed RSV and RBSDV infection activated the JAK-STAT pathway and promoted the accumulation of SOCS5, and accelerated the BCL2 degradation through the 26S 30 proteasome pathway, causing the activation of apoptosis facilitated persistent viral infection in their vector. This is an interesting story about virus-vector interaction and is well-written. I would recommend acceptance after responding to the listed concerns.*

**Response: We greatly appreciate your interest and positive comments to our work. The manuscript has been carefully modified and improved following your suggestions.**

*Reviewer #2: This is a resubmitted manuscript. The authors have address all of my previous comments.*

**Response: Thanks for your positive and valuable comments.**

*Reviewer #3: In this manuscript authors described the role of JAK-STAT pathway in promoting persistent viral infection by activating apoptosis in insect vectors small brown planthopper. They showed that both RSV and RBSDV, two important rice viruses transmitted by small brown planthopper, activated the JAK-STAT pathway and promoted the accumulation of Suppressor of cytokine signaling 5 (SOCS5), an E3 ubiquitin ligase regulated by the JAK-STAT transcription factor STAT5B. The manuscript is significantly improved after reversion.*

**Response: Thanks for your great comments and encouragement.**

*Major Issues:*

*Reviewer #1: 1, The evidences of SOCS5 interacts and degrade BCL2 are relatively less, and there is only on data (Fig 3E) showed how BCL2 was degraded. Is it possible to show BCL2 was ubiquitized by SOCS5 and the ubiquitination is enhanced by virus infection.*

**Response: Thanks for your good suggestions. As an E3 ubiquitin ligase, SOCS5 is responsible for degradation of BCL2. We will test the possibility that virus infection can enhance the host ubiquitination in further research.**

*2, In Fig 4E and 4G, in 1 dpi, the transcript level of SOCS5 was significantly increased, but the protein level was similar. And in Fig 4F, the PCR showed the RSV level was accumulated at 1, 2, and 4 dpi, but in Fig 4E, RSV-NP was only detected in 4 dpi.*

**Response: Thank you for your suggestions. Although the transcript level of SOCS5 was increased in 1 dpi, the protein levels of SOCS5 were increased at 2 dpi and 4 dpi. One possible explanation for this phenomenon is that it could take time for SOCS5 protein accumulation from transcription to translation. For RSV accumulation, qRT-PCR has higher sensitivity than western blotting, which is difficult to detect in the early stage of virus infection.**

*3, Some immunoblot of phos-STAT5B was low quality, like in Fig 4D and 4E, one has a specific one band, and the other has two bands.*

**Response: We have replaced the high quality images (new Fig 4D).**

*Minor Issues:*

*Reviewer #1: 1, There is only Fig 6 showed RBSDV may has similar mechanism, the data is relatively weak and I think the authors has over-stated the conclusion.*

**Response: Thanks for point it out. Similar to the results of RSV infection, the BCL2 accumulation was reduced, the protein levels of P-STAT5B and SOCS5 increased during RSV infection. Also, knockdown of two key genes (*STAT5B* and *SOCS5*) in the JAK-STAT pathway significantly inhibited RBSDV accumulation in SBPHs (Fig 6). Thus, JAK-STAT pathway may also promote RBSDV infection.**

*2, Line 235, there are two “STAT5B”, please delete one.*

**Response: Thanks for your suggestions. We have revised it (new Line 235).**
